# Transcriptome Analysis of Potato Leaves under Oxidative Stress

**DOI:** 10.3390/ijms25115994

**Published:** 2024-05-30

**Authors:** Juping Liu, Xun Tang, Huanhuan Zhang, Meng Wei, Ning Zhang, Huaijun Si

**Affiliations:** 1 State Key Laboratory of Aridland Crop Science, Gansu Agricultural University, Lanzhou 730070, China; 15139420026@163.com (J.L.); tangxun@gsau.edu.cn (X.T.); 18298496342@163.com (H.Z.); ningzh@gsau.edu.cn (N.Z.); 2 College of Life Science and Technology, Gansu Agricultural University, Lanzhou 730070, China; 15349877760@163.com; 3 College of Agronomy, Gansu Agricultural University, Lanzhou 730070, China

**Keywords:** *Solanum tuberosum* L., oxidative stress, transcriptome, differentially expressed genes, reactive oxygen species

## Abstract

Potato (*Solanum tuberosum* L.) is a major global food crop, and oxidative stress can significantly impact its growth. Previous studies have shown that its resistance to oxidative stress is mainly related to transcription factors, post-translational modifications, and antioxidant enzymes in vivo, but the specific molecular mechanisms remain unclear. In this study, we analyzed the transcriptome data from potato leaves treated with H_2_O_2_ and Methyl viologen (MV), and a control group, for 12 h. We enriched 8334 (CK vs. H_2_O_2_) and 4445 (CK vs. MV) differentially expressed genes (DEGs), respectively, and randomly selected 15 DEGs to verify the sequencing data by qRT-PCR. Gene ontology (GO) enrichment analysis showed that the DEGs were mainly concentrated in cellular components and related to molecular function, and Kyoto Encyclopedia of Genes and Genomes (KEGG) enrichment analysis indicated that most of the DEGs were related to metabolic pathways, plant hormone signal transduction, MAPK-signaling pathway, and plant–pathogen interactions. In addition, several candidate transcription factors, mainly including MYB, WRKY, and genes associated with Ca^2+^-mediated signal transduction, were also found to be differentially expressed. Among them, the plant hormone genes Soltu.DM.03G022780 and Soltu.DM.06G019360, the CNGC gene Soltu.DM.06G006320, the MYB transcription factors Soltu.DM.06G004450 and Soltu.DM.09G002130, and the WRKY transcription factor Soltu.DM.06G020440 were noticeably highly expressed, which indicates that these are likely to be the key genes in the regulation of oxidative stress tolerance. Overall, these findings lay the foundation for further studies on the molecular mechanisms of potato leaves in response to oxidative stress.

## 1. Introduction

Plants are subjected to a variety of stresses during growth, including biotic and abiotic stresses [1]. Abiotic stresses mainly include drought stress, high-salt stress, and high- and low-temperature stress, all of which can lead to the accumulation of excess ROS in plants, resulting in oxidative stress damage, aggravating the degree of membrane lipid oxidation and severely inhibiting plant growth [2]. Plants respond to oxidative stress through complex physiological and biochemical processes, including sensing and recognition of stress signals, activation of pathways during stress, and changes in the regulation of stress-related gene expression. As a major food crop worldwide, the potato is rich in nutritional value [3]. The study of the molecular mechanisms of its response to oxidative stress further elucidates the effects of oxidative stress on its growth and development and provides a reference for the improvement of high-quality potato varieties.

Methyl viologen (MV), also known as Paraquat dichloride [4], is an inducer that has been widely used in the study of plant oxidative stress tolerance [5]. The exogenous application of MV to the plant chloroplast photosystem I and mitochondrial complexes I and III can provide a large number of electrons for it, which produces O_2_^•−^, which is extremely unstable and easily dismutated to H_2_O_2_ by superoxide dismutase (SOD) [6]. H_2_O_2_ is one of the most abundant ROS in the cell and is closely related to the plant response to abiotic stresses [7]. Studies have shown that H_2_O_2_ can interact with a variety of plant hormone signaling pathways, such as salicylic acid (SA), abscisic acid (ABA), and auxin (IAA), thereby causing a series of gene expression changes in response to stress [8,9].

The intracellular environment of the plant is in a reduced state during normal growth, and a large number of ROS will be generated when the plant suffers from stress. These ROS mainly include single oxygen (^1^O_2_), superoxide anion (O^−^ _2_), hydrogen peroxide (H_2_O_2_), and hydroxyl radical (HO^.^), which are very active and highly reactive [10]. The accumulation of excessive ROS will lead to the destruction of the redox state in the cell and will have a poisonous effect on the cell [11]. When plants are subjected to MV and H_2_O_2_ treatment, a large amount of ROS is produced in the cells. These ROS act as signaling molecules, along with Ca^2+^, MAPK, and some transcription factors, to participate in the plant’s response to adversity [12]. This suggests that various metabolic and signaling pathways are necessary to respond to oxidative stress [13,14]. Moreover, the plant cells will scavenge excessive ROS to maintain the dynamic balance of ROS. The systems for scavenging ROS in plants are mainly categorized into enzymatic and non-enzymatic systems. The enzymatic system mainly includes Superoxide dismutase (SOD), Catalases (CAT) and Peroxidase (POD), Glutathione S-transferases (GST), Ascorbate peroxidase (APX), and Glutathione peroxidase (GPX). The non-enzymatic system consists of antioxidants such as Flavonoid, Glutathione, and Carotene [15]. It has been demonstrated that, under exogenous MV treatment, the overexpression of the GLDH gene, a key enzyme in rice ascorbate synthesis, significantly improved the antioxidant capacity and overexpression of SOD and APX in sweet potato and enhanced its tolerance to oxidative stress [16]. Overexpression of SOD and APX can enhance the tolerance of transgenic tobacco to MV [17].

A current transcriptome analysis of plant resistance to abiotic stress shows that signal transduction, protease, plant hormones, transcription factors, and so on, play an important role. However, studies on the mechanism of the oxidative stress response in plants subjected to MV and H_2_O_2_ stress are still shallow, and most of them focus on the study of antioxidant enzymes in plants. In this study, MV and H_2_O_2_ were uniformly sprayed on potato plant leaves and samples were collected 12 h later for transcriptome sequencing. Analysis of the sequencing results showed that the DEGs were mainly concentrated in the plant–pathogen interaction, MAPK signaling pathway, and plant hormone signaling pathway. In addition, we also identified some DEGs related to transcription factors and Ca^2+^ signaling. This provides us with an effective resource to study the molecular mechanisms of oxidative stress tolerance in potato plants.

## 2. Results

### 2.1. Antioxidant Enzyme Activities of Potato under Oxidative Stress

**Figure 1:**
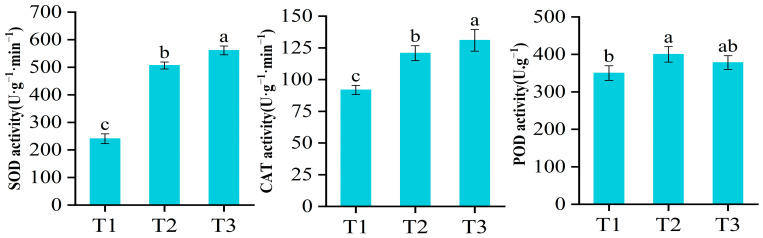
Physiological indexes of potato leaves’ SOD, CAT, and POD activities were determined under oxidative stress. The values are means ± SD of three replicates (*p* < 0.05). The a, b, and c represent significant differences.

In the present study, changes in the SOD, CAT, and POD activities of potato leaves were determined after treatment with 30 μM MV and 20% H_2_O_2_ for 12 h. Among them, the activities of SOD and CAT were significantly higher in the treated group than in the control group, while the changes in the activity of POD showed a slight increase (Figure 1). The alterations in the activities of these enzymes were closely associated with ROS scavenging. Therefore, it is evident that the surplus ROS were eliminated by enhancing the activities of SOD, CAT, and POD under oxidative stress.

### 2.2. Overview of RNA Sequencing and Mapping

A total of 1.26 million (T1), 1.70 million (T2), and 1.74 million (T3) clean reads were generated from the transcriptome libraries. The average number of clean reads mapped to the reference genome for T1, T2, and T3 was 1.04 (81.98%), 1.10 (85.94%), and 1.04 (81.1%). Overall, about 75.53–93.79% of clean reads were mapped to the reference genome (Table 1).

**Figure 2:**
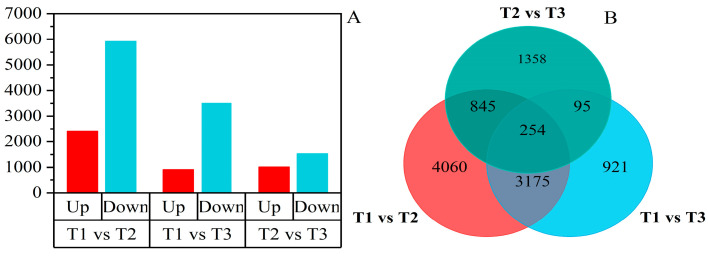
Differentially expressed genes (DEGs) analysis. (**A**) The number of up- and down-regulated DEGs among T1 vs. T2, T1 vs. T3, and T2 vs. T3 comparisons. (**B**) Venn diagram comparison of the number of DEGs.

The number of DEGs between T1 and T2 was 8334, with 2412 up-regulated genes and 5922 down-regulated genes. The number of DEGs between T1 and T3 was 4445, with 915 up-regulated genes and 3530 down-regulated genes. In addition, the number of DEGs between T2 and T3 was analyzed to be 2552, with 1015 up-regulated genes and 1537 down-regulated genes (Figure 2A). The Venn diagram of DEGs indicated that 3430 genes were common to both stress treatments (Figure 2B). These results suggest that most genes were regulated during oxidative stress.

### 2.3. GO Annotation and Enrichment

**Figure 3:**
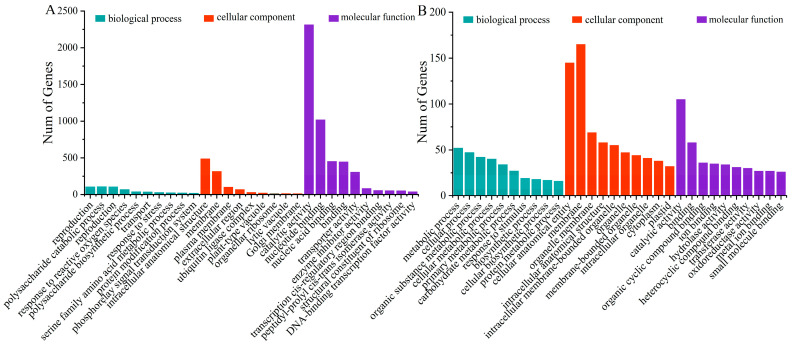
GO classification analysis of DEGs in (**A**) T1 vs. T2 and (**B**) T1 vs. T3.

To better study the functions of DEGs in potato leaves under oxidative stress, GO annotations were analyzed for T1 and T2, as well as T1 and T3. The top 10 enriched categories were analyzed in each component. The functional properties of DEGs were mainly related to “membrane”, “catalytic activity”, “nucleotide binding”, and “binding”. This indicates that DEGs involved in these processes play a significant role in the oxidative stress of potato plants (Figure 3).

### 2.4. KEGG Analysis of DEGs

**Figure 4:**
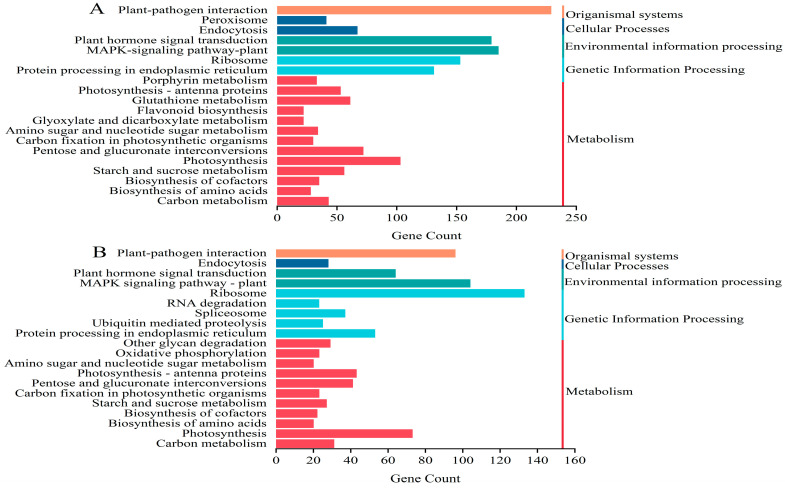
KEGG enrichment analysis of DEGs in (**A**) T1 vs. T2 and (**B**) T1 vs. T3.

KEGG is primarily used to predict the functions of expressed genes. To gain a deeper understanding of the functions of DEGs, a KEGG enrichment analysis was performed on them. The top 20 results of the enriched entries revealed that the DEGs under the two stress treatments were primarily associated with the “plant–pathogen interaction”, “MAPK-signaling pathway-plant”, “ribosome”, and “plant hormone signal transduction”. The most significant enrichment was observed in the metabolic pathway, which also encompassed “photosynthesis” and the synthesis of secondary metabolites (Figure 4). These pathways are associated with plant responses to abiotic stresses, and mitogen-activated protein kinase (MAPK) plays an important role in regulating plant growth and stress tolerance. It is also a significant regulator of the plant antioxidant system in response to various stimuli. The plant hormone signaling pathway is crucial for responding to stress and throughout the growth and development of a plant. At different stages of plant growth, a variety of plant hormones act synergistically or antagonistically to guide the normal growth of plants, including adaptation to the environment and resistance to stress.

### 2.5. Expression Changes in ROS Scavenging-Related Genes

**Figure 5:**
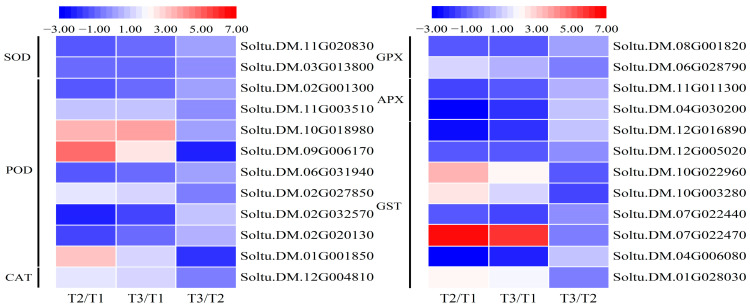
Heatmaps of EDGs encoding genes about SOD, POD, CAT, GPX, APX, and GST. Each column represents the mean expression value (log_2_ FPKM, T2 sample is divided by T1 and T3 sample is divided by T1 and T2, respectively) of three biological replicates obtained from RNA-Seq data.

Oxidative stress induces the production of large amounts of ROS in potato plants. Therefore, enzymatic and non-enzymatic systems in the plant are required to scavenge the excessive accumulation of ROS. It has been shown that SOD, POD, CAT, GPX, APX, and GST are the key enzymes for scavenging ROS in plants. Among them, SOD, POD, and CAT are the most common classes of ROS-scavenging enzymes. Under adverse stress conditions, the expression of plant genes encoding these enzymes increased significantly, enhancing the scavenging ability of ROS. Consequently, the resistance of plants to adverse environments could be improved. In this experiment, a total of two SOD genes, eight POD genes, and one CAT gene were differentially expressed. Compared with the control group, the expression levels of a total of six genes increased. Among them, two genes (Soltu.DM.09G006170 and Soltu.DM.10G018980) showed the most obvious changes, with a 4.7-fold and 3.3-fold increase under the H_2_O_2_ treatment, and approximately a 2.4-fold and 3.6-fold increase under the MV treatment (Figure 5).

GPX can effectively scavenge cellular peroxides and prevent the cellular damage caused by excess ROS, which plays an important role in plant response to adversity stress. The expression of plant GPX genes is complex. In the present study, a total of two GPX genes were differentially expressed. Except for Soltu.DM.06G028790, the other one showed significantly down-regulated expression compared to the control. APX expression is induced by various biotic and abiotic stresses. In this study, a total of two APX genes were differentially expressed compared to the control, and both of them exhibited down-regulated expression. This down-regulation is speculated to be possibly associated with the concentration and the duration of treatment with H_2_O_2_ and MV. GST has a wide range of functions and participates in cellular processes such as detoxification, antioxidant activity, signal transduction, and more. This study identified that a total of eight GST genes were differentially expressed, four of which were up-regulated (the highest by 6.5-fold under H_2_O_2_ treatment and 5.8-fold under MV treatment) and four of which were down-regulated (the highest by 2.8-fold under H_2_O_2_ treatment and 2.1-fold under MV treatment) (Figure 5). This suggests that the mechanisms by which antioxidants function in plants are complex and can help mitigate the damage caused by stress to plants.

### 2.6. Expression Changes in Plant Hormone Signalling

**Figure 6:**
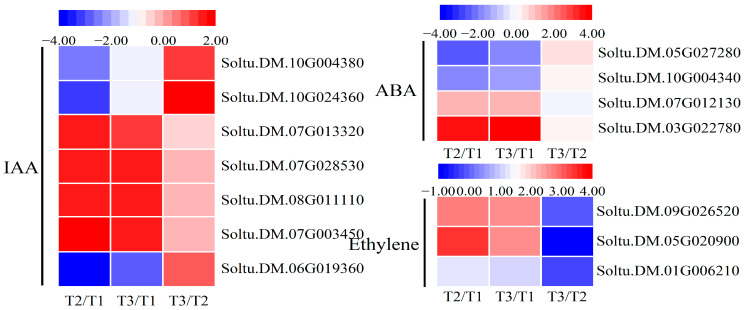
Heatmaps of EDGs involved in plant hormone signaling, including IAA, ethelene, and ABA. Each column represents the mean expression value (log_2_ FPKM, T2 sample is divided by T1, T3 sample is divided by T1 and T2, respectively) of three biological replicates obtained from RNA-Seq data.

Plant hormones often form a complex interaction network to regulate various physiological activities of plants, especially playing a key role in plant defense against abiotic stress. Auxin (IAA), the first plant hormone discovered, is synthesized in the tender parts of the plant and transported to target organs through both polar and non-polar transport to play its roles in regulating the growth and development of the plant. Many gene families play a crucial role in the signaling pathway of IAA, such as Aux/IAA, GH3, and SAUR. A total of three *Aux/IAA*, three *SUAR*, and one *GH3* DEG were detected by sequencing, all of which showed up-regulated expression under oxidative stress, except for Soltu.DM.06G019360, Soltu.DM.10G024360, and Soltu.DM.10G004380 (Figure 6). This suggests that IAA has complex functions in potato plants under oxidative stress.

Under normal conditions, the ethylene content in plants remains low. However, when subjected to stress, the content changes significantly. The ethylene produced in response to stress stimuli signals will be transmitted downstream through the corresponding signal-transduction pathway and will regulate downstream genes, thereby triggering a series of reactions in plant cells. Transcriptome analysis showed that there were three differentially expressed ethylene-related genes, all of which were up-regulated. Among them, the expression of Soltu.DM.05G020900 was the most significant, showing a 3.5-fold and 2.6-fold increase compared to the control group, respectively (Figure 6). Abscisic acid (ABA) plays a wide range of roles in plants and is involved in regulating seed dormancy and germination, leaf senescence, organ abscission, root development, and stress response. The core components of the ABA signaling pathway are PYR/PYL proteins, PP2C phosphatase, SnRK2 kinase, and ABF/AREB transcription factors. In this study, one PYR/PYL gene (Soltu.DM.03G022780), two PP2C genes (Soltu.DM.07G012130 and Soltu.DM.10G004340), and one SnRK2 gene (Soltu.DM.05G027280) were enriched under oxidative stress. The up-regulated expression of the PYR/PYL gene was the most obviously, 3.5-fold and 3.8-fold higher than that of the control (Figure 6). In summary, these hormones are involved in the regulation of oxidative stress tolerance in potato plants. However, their complex expression patterns and regulatory mechanisms require further study.

### 2.7. Genes Involved in Ca^2+^ Signal Pathway

**Figure 7:**
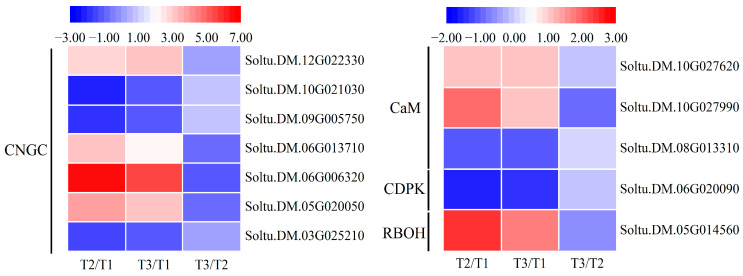
Heatmaps of DEGs encoding genes related to Ca^2+^ signal transduction during oxidative stress of potato plants. Each column represents the mean expression value (log_2_ FPKM, T2 sample is divided by T1, T3 sample is divided by T1 and T2, respectively) of three biological replicates obtained from RNA-Seq data.

Cyclic nucleotide-gated channel proteins (CNGCs) are in the Ca^2+^ transport channel family and play important roles in plant development, light, salt, abiotic stress response, and signal transduction. According to transcriptome data, a total of seven CNGC genes were enriched, and four of them were up-regulated, with the highest expressions being 6.6-fold and 5.6-fold higher (Soltu.DM.06G006320) (Figure 7). In addition, the expressions of three calcium-binding proteins (CaM) were also enriched, of which the gene Soltu.DM.10G027990 showed the highest up-regulation in both treatments compared to the control, with 1.9-fold and 1-fold increases, respectively (Figure 7). The phosphorylation cascades regulated by kinases are the main downstream reactions of Ca^2+^ signaling. Calcium signals are sensed and transmitted by Ca^2+^-dependent kinases and phosphatases. The calcium-dependent protein kinases (CDPKs) family, as unique calcium-dependent protein kinases in plants, co-regulate cell homeostasis along with other kinases. In this experiment, one CDPK gene was enriched and showed down-regulated expression, suggesting that this CDPK gene negatively regulates potato resistance to oxidative stress.

Respiratory burst oxidase homologs (RBOHs) are one of the key enzymes for ROS production in plants. When plants are stimulated by the external environment, the concentration of cytoplasmic free Ca^2+^ increases. The NADPH oxidase RBOHD/F located on the membrane is activated by BIK1 and CDPKs at different phosphorylation sites of Ca^2+^ to produce ROS. Meanwhile, ROS can induce Ca^2+^ production. In the transcriptome data, one RBOH gene was enriched, and it showed up-regulated expression (Figure 7). This suggests that the potato oxidative stress signal stimulated an increase in RBOH content, leading to a large amount of ROS production and triggering a series of stress responses.

### 2.8. Expression Changes in Transcription Factors

**Figure 8:**
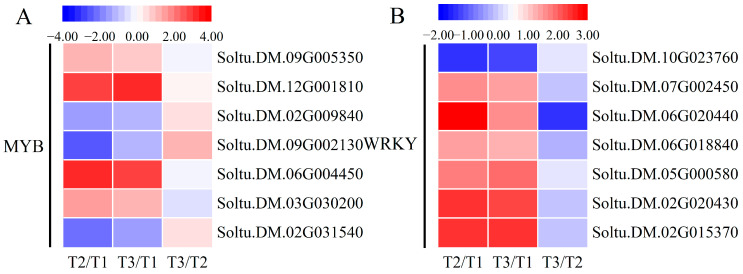
Heatmaps of DEGs encoding transcription factors. (**A**) The transcription factor of MYB. (**B**) The transcription factor of WRKY. Each column represents the mean expression value (log_2_ FPKM, T2 sample is divided by T1, T3 sample is divided by T1 and T2, respectively) of three biological replicates obtained from RNA-Seq data.

Many transcription factor families exist in plants, such as MYB, WRKY, NAC, and bZIP. In this study, we mainly enriched MYB and WRKY transcription factors. MYB transcription factors are involved in many abiotic stress pathways in plants. In this study, a total of seven MYB transcription factors were enriched, with three being down-regulated (Soltu.DM.02G031540, Soltu.DM.09G002130 and Soltu.DM.02G009840) and the remaining four being up-regulated (Figure 8A). It can be seen that MYB transcription factors play a complex role in oxidative stress. WRKY transcription factors are one of the largest families of transcription factors in plants and are important components of plant adversity stress signaling pathways. According to the sequencing results, seven WRKY transcription factors were enriched, all of which exhibited up-regulation of expression except for Soltu.DM.10G023760. The highest up-regulated expression was 3.0-fold and 1.5-fold (Figure 8B). The results indicate that most WRKY transcription factors induced by H_2_O_2_ and MV may positively regulate the ability of potato plants to cope with oxidative stress, which is conducive to future studies.

### 2.9. Verification of RNA-Seq Data by Quantitative Real-Time PCR (qRT-PCR)

**Figure 9:**
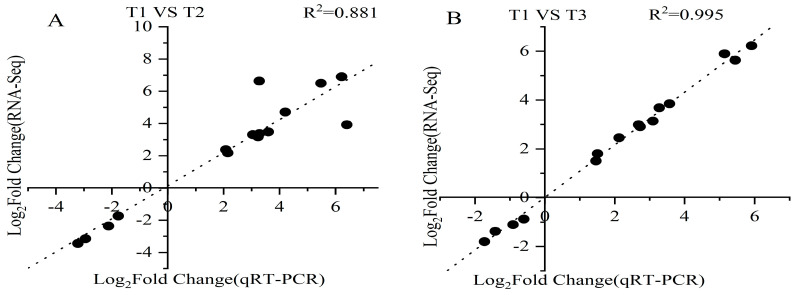
Verification of RNA-Seq results with qRT-qPCR in (**A**) T1 vs. T2 and (**B**) T1 vs. T3.

To verify the reliability of the transcriptome data, fifteen DEGs were randomly selected for qRT-PCR, including eleven up-regulated genes and four down-regulated genes. The fold changes of the genes between the two oxidative stress treatments and the control were compared with the RNA-Seq data, and the data were analyzed for correlation. The results showed that the data derived from qRT-PCR were consistent with the expression trend of the RNA-Seq data. The correlation coefficient was 0.881 for the H_2_O_2_-treated group compared with the control, and 0.995 for the MV-treated group compared with the control. This indicates that the transcriptome data were highly reliable (Figure 9).

## 3. Discussion

When plants are subjected to abiotic stresses, the intracellular redox homeostasis is disrupted, which further induces secondary oxidative stress damage. In the process of studying the mechanisms regulating oxidative stress tolerance in potato plants, we utilized RNA-Seq to identify changes in potato leaf transcripts under two different oxidative stress conditions. A total of 8334 and 4445 DEGs were identified compared to the control group, which provided the foundation for a more in-depth analysis of DEGs.

ROS are produced and accumulated in large quantities in plants under oxidative stress [18]. These excess ROS can disrupt intracellular homeostasis and have toxic effects on cells [19]. To minimize the oxidative damage caused by these ROS, plants have a self-protection system for scavenging ROS, which mainly consists of enzymatic and non-enzymatic systems. In this study, we measured the activities of three common antioxidant enzymes: SOD, CAT, and POD. We found that the activities of all three enzymes increased under oxidative stress. It can be seen that the enzyme system, which can quickly scavenge ROS, plays a key role in protecting plants from adversity. At the same time, we also analyzed the expression of several common ROS-scavenging enzymes in the transcriptome data. Among these, the number of DEGs related to POD and GST was the highest. Of course, the stress-induced protection mechanism is complex. It is not only these enzymes that play a protective role, but also other antioxidants and signaling molecules that work together to protect the plant from oxidative stress. The interaction between regulatory networks needs further study.

In addition to primary abiotic stress signals, plants also require secondary oxidative stress signals during growth, which play an important role in the plant’s response to oxidative stress [20,21]. Several studies in recent years have shown that ROS can integrate with different environmental signals or activate stress response pathways to help plants enhance stress tolerance [22,23,24]. Previous studies have confirmed that ROS act as a second messenger in ABA signal transduction, but the molecular mechanism is not yet clear. ABA is a prevalent hormone in plants, and early studies have shown that exogenous ABA treatment can increase POD activity in tobacco under salt stress and reduce the damage caused by H_2_O_2_ [25]. In this study, we identified some genes related to the ABA signaling pathway under oxidative stress, such as PYR/PYL, PP2C, and SnRK2. The PYR/PYL gene showed the highest expression, suggesting that this gene may positively regulate tolerance to oxidative stress in potato plants.

When the external environment changes, the superoxide anion produced by NADPH oxidase in the plant plasma membrane is disproportionated to H_2_O_2_ [26]. This process allows Ca^2+^ to flow inward, thereby activating intracellular calcium signaling and downstream signal transduction processes [27,28]. It has been shown that HPCA1, a leucine-rich repeat receptor kinase located on the cell surface in Arabidopsis, is a receptor for extracellular H_2_O_2_ and is involved in the H_2_O_2_-induced activation of calcium signaling [29]. CNGC is an ion transport channel for calcium, which is important for calcium uptake and transport in plants and regulates the plant response to biotic and abiotic stresses [30]. However, free calcium does not function directly in plants, it needs to bind to calcium ion sensors for downstream regulation, so CaMCML and CDPK play important roles in calcium signaling [31,32]. In the transcriptome data, we enriched a total of eight *CNGC* genes, three *CaMCML*, and one *CDPK* gene, and the differential expression of these genes is closely related to plant calcium signaling in response to oxidative stress.

In addition, the plant’s own antioxidant defenses during oxidative stress are also regulated by a number of protein kinases and transcription factors [33]. Protein kinases, such as MAPK, are key regulators that modulate the antioxidant defenses and response to stress [34,35]. Oxidative stress induces phosphorylation of MAPK by H_2_O_2_, which then participates in a signaling cascade that regulates the expression of downstream genes [36]. In this study, there were three DEGs of MAPK, and all of them were up-regulated (Table 2), suggesting that oxidative stress induces a phosphorylation cascade in MAPK. The phosphorylation cascade in plants regulates gene expression by modulating the activity of transcription factors, which ultimately improves plant adaptation to stress [37]. Studies have shown that activated MAPK6 in *Arabidopsis* interacts with MYB transcription factors to improve plant salt tolerance [38]. MYB and WRKY, as two types of transcription factors with numerous families in plants, are involved in regulating multiple signaling pathways. *XsWRKY20* in *Xanthoceras sorbifolium* positively regulates drought stress tolerance by integrating ROS homeostasis and ABA signaling pathways to regulate antioxidant enzyme-related genes [39]. However, overexpression of *CbWRKY27* in *Catalpa bungei*, integrating the ABA and ROS pathway, resulted in plant sensitivity to ABA, decreased antioxidant enzyme activity, and increased H_2_O_2_ content negatively regulates salt tolerance [40]. Arabidopsis *AtMYB30* can participate in signaling and the regulation of salt, heat, and oxidative stress with ROS and calcium signaling pathways [41]. These findings suggest that the role of transcription factors in the downstream regulation of ROS is complex. In this study, we focused on analyzing some MYB and WRKY transcription factors in potato plants under oxidative stress. Most of the transcription factors showed up-regulated expression, of these, Soltu.DM.06G004450 and Soltu.DM.06G020440 had the highest expression. This indicates that these two transcription factors may positively regulate tolerance to oxidative stress in potato plants.

**Figure 10:**
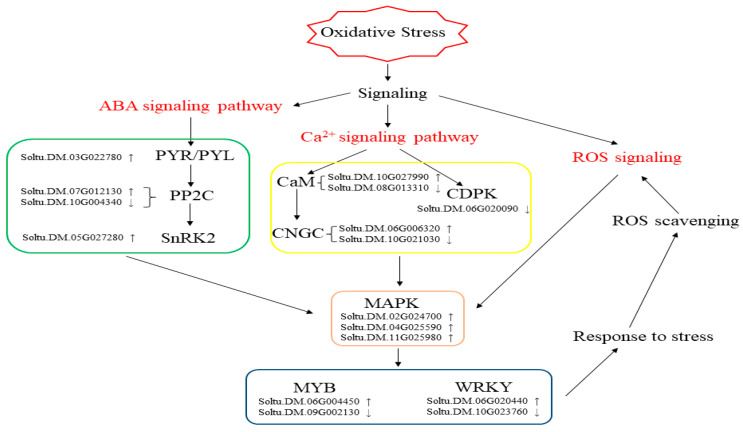
The model of pathways in response to oxidative stress (↑ representative up regulation; ↓ representative down regulation).

In this paper, we analyzed transcriptome data to explore the pathways involved in the response of potato leaves to oxidative stress. Based on the transcriptome data, a model of the response to oxidative stress was proposed (Figure 10). When plants perceive oxidative stress it is transmitted downstream through Ca^2+^, plant hormones, and ROS signaling, ultimately leading to changes in intracellular metabolism. These signaling pathways interact with protein kinases to activate downstream transcription factors. This series of cascading reactions allows plants to respond to oxidative stress.

## 4. Materials and Methods

### 4.1. Plant Material and Oxidative Stress Treatment

The potato variety “Atlantic” was used as the research material. Potato tubers were planted in 10 cm × 10 cm pots (nutrient soil: vermiculite = 3:1) and incubated for 30 days at 24 °C, a light intensity of 2000 Lx, and a cycle of 16 h of light/8 h of darkness. After that, healthy plants with uniform growth status were selected for subsequent experiments. A total of 30 μM MV (T2) and 20% H_2_O_2_ (T3) was uniformly sprayed on the second to third leaves at the top of potato plants in the treatment group; the control group (T1) was uniformly sprayed with distilled water, three plants from each group of treatments were selected as biological replicates, and the samples were taken after 12 h of treatment. All samples were frozen in liquid nitrogen and stored at −80 °C for measuring physiological indexes and were sent to the company for transcriptome sequencing.

### 4.2. GO and KEGG Pathways Enrichment Analysis

Within-group differential gene analysis was performed using DESeq under the conditions of |Log_2_ Fold Change| ≥ 1 and adjusted *p*-value ≤ 0.01. PossionDis was used to perform a between-group differential gene analysis under the conditions of |Log_2_ Fold Change| ≥ 1 and FDR ≤ 0.001. The heatmap function on the differential gene set was used to draw a heatmap of differential gene clusters. According to the GO and KEGG annotation results and classifications, the DEGs were functionally classified, the phyper in R-4.2.2 software was used for KEGG enrichment analysis, and the TermFinder package was used for GO Enrichment analysis (https://metacpan.org/pod/GO::TermFinder, accessed on 1 June 2023). With a Q value of ≤0.01 as the threshold, candidate genes that met this condition were defined as significantly enriched.

### 4.3. Measurement of Antioxidant Enzyme Activities

SOD activity was determined after fully grinding the preserved leaves with liquid nitrogen using the method described by Giannopplitis and Ries et al. [42]. CAT activity was determined by the decomposition of H_2_O_2_ using the method described by Aebi [43], and POD activity was determined using the method described by Maehly and Chance [44]. In the above experiments, three plants were selected from each group, and each plant was subjected to three biological replications and three technical replicates.

### 4.4. RNA Extraction and RNA-Seq

RNA extraction and cDNA library construction, as well as transcriptome sequencing, were performed by the Beijing Genomics Institution using the DNBSEQ platform. The raw data obtained from sequencing were filtered to remove low-quality reads and those with high base N content to obtain clean reads, which were then aligned to the reference genome sequence. The software calculated the fragments per kilobase of transcription per million mapped reads (FPKM) and then analyzed the DEGs using DESeq. DEGs were considered significant if they met the thresholds of |log_2_ Fold change| ≥ 1 and *p*-values ≤ 0.01. The raw sequence data reported in this paper have been deposited in the Genome Sequence Archive (Genomics, Proteomics & Bioinformatics 2021) in the National Genomics Data Center (Nucleic Acids Res 2022), China National Center for Bioinformation/Beijing Institute of Genomics, Chinese Academy of Sciences (GSA: CRA014950), and are publicly accessible at https://ngdc.cncb.ac.cn/gsa (accessed on 18 February 2024).

### 4.5. Validation of Differentially Expressed Genes by Quantitative Real-Time PCR (qRT-PCR)

To ensure the reliability of the RNA-Seq results, 15 DEGs were randomly selected for qRT-PCR. The gene IDs in the PGSC database and their specific primers are provided in Supplementary Table S1. The plant RNA and cDNA were extracted and synthesized following the instructions of the TIANGEN Total RNA Extraction Kit and FastKing RT Kit (with gDNase). qRT-PCR was performed on a Light Cycler 96 SW 1.1 system in a reaction mixture volume of 20 μL containing 1 μL cDNA, 10 μL 2 × Universal Blue SYBR Green qPCR Master Mix, 1 μL forward primer, 1 μL reverse primer, and 7 μL RNase-Free ddH_2_O. Gene expression was standardized using elongation factor 1-alpha 1 (ef1a). The reaction conditions were as follows: 94 °C for 30 s, followed by 40 cycles at 95 °C for 15 s and 60 °C for 30 s. The 2^−ΔΔCt^ method was chosen to calculate the relative fold change in template abundance for each sample [45].

### 4.6. Statistical Analysis

The statistical analysis was performed using Microsoft Excel 2016 and SPSSS 25. Charts were drawn by Origin Pro 2024 (10.1) software. The Kolmogorov–Smirnov normality test and Levene’s homogeneity of variance test were used for evaluation before the statistical analysis. Results were analyzed using data variance analysis performed with the ANOVA Duncan’s test. *p* < 0.05 was defined as indicating significant difference.

## 5. Conclusions

This study investigated pathways that are involved in the response of potato leaves to oxidative stress. Genes were categorized by GO and KEGG analysis. The data indicated that Ca^2+^, plant hormones, and transcription factors were all involved in oxidative stress. Among them, the expression of the CNGC gene Soltu.DM.06G006320, which is related to Ca*^2+^* transport, was significantly changed, suggesting that Ca^2+^ signaling has an important role in the response to oxidative stress. These signals activate downstream transcription factors (such as the Soltu.DM.06G004450 transcription factor, with significant changes in expression) through the protein kinase pathway, resulting in changes in gene expression in response to stress.

## Figures and Tables

**Table 1:** The statistics of reads and the mapped reads compared with the genome.

Samples		Raw Reads	Clean Reads	Mapped Reads	Mapped Ratio (%)	Q30 (%)
**T1**	T1-1	43,684,236	42,252,294	39,628,427	93.79	95.28
	T1-2	43,684,592	42,095,838	32,081,238	76.21	95.77
	T1-3	43,684,838	42,041,882	31,926,605	75.94	95.63
**T2**	T2-1	43,683,506	42,190,804	36,625,837	86.81	94.81
	T2-2	43,684,728	42,244,960	35,097,113	83.08	95.01
	T2-3	45,432,984	43,560,802	38,298,658	87.92	95.91
**T3**	T3-1	43,676,094	42,216,594	35,048,217	83.02	93.89
	T3-2	45,432,568	43,568,446	34,568,446	79.93	95.43
	T3-3	43,684,752	42,283,118	33,957,573	80.31	95.44

**Table 2:** DEG information of MAPK.

Gene ID	Description	Log_2_T2/T1	Log_2_T3/T1
Soltu.DM.02G024700	mitogen-activated protein kinase 7/14	1.99	1.88
Soltu.DM.04G025590	mitogen-activated protein kinase 3	1.69	1.66
Soltu.DM.11G025980	mitogen-activated protein kinase 1/3	1.56	1.21

## Data Availability

The original contributions presented in this study are included in the article/Supplementary Materials, further inquiries can be directed to the corresponding author.

## Supplementary Materials

The following supporting information can be downloaded at: https://www.mdpi.com/article/10.3390/ijms25115994/s1.
