# Supplementary material for: Transcriptome Analysis of Potato Leaves under Oxidative Stress
Source: Int J Mol Sci. 2024 May 30;25(11):5994. doi: 10.3390/ijms25115994 (PMC11172952; doi:10.3390/ijms25115994)
Supplement: Supplementary file 1 [file ijms-25-05994-s001.zip › ijms-2981192-supplementary.pdf]

Supplementary Table S1. Primers used for qRT-PCR to validate the correlation of RNA-seq

Gene ID	Description	Primers for qRT-PCR (5′-3′)		Log2(T2/T1)	Log2(T3/T1)
Soltu.DM.05G014560	Respiratory burst oxidase	GCATTAGTGCGAAGAAGGCG	ATCTCAAGCCTGGCGTGAAA	2.38	1.80
Soltu.DM.05G020050	Cyclic nucleotide gated channel, plant	ATGGCAAGAAACCAGGGGAG	AGTGCTGGCTTCAGTCTGTC	3.92	3.14
Soltu.DM.06G006320	Cyclic nucleotide gated channel, plant	TTTATGGTGACGCGGTGACA	CAAAAAGAACCAGGCCGAGC	6.64	5.63
Soltu.DM.06G004450	Transcription factor MYB, plant	ACGCGTCTAGTATTTCTCCATCT	TGGGCGTGAAATCCACATCA	3.31	2.99
Soltu.DM.03G022780	Abscisic acid receptor PYR/PYL family	TCAAGCACATTCGAGCACCT	GGTACTCGTTGTTGCAGGGA	3.48	3.85
Soltu.DM.05G027280	Serine/threonine-protein kinase SRK2	CCAGTCCTGCCAGGAGAATC	AACTGGCCTTGATGGAGGTG	-2.37	-1.80
Soltu.DM.10G024360	SAUR family protein	CCAAAGGAAGAGCCAAAGAGG	TCAGTGTGTAAGGCTGACGC	-3.15	-1.10
Soltu.DM.09G006170	Peroxidase	TCCACAGGCTTTACCCAACG	GTTTGGTGACGTGCATCGAC	4.72	2.45
Soltu.DM.10G018980	Peroxidase	TCACATTGCAGCTCGTGACT	GGAATATTGTCGTTGGCCGC	3.37	3.69
Soltu.DM.07G022470	glutathione S-transferase	ACAAGCTAGGTTCTGGGCTG	TACCTGCCTCCTGCTCTTCT	6.50	5.89
Soltu.DM.12G029660	Ubiquitin-conjugating enzyme E2 O	CAAAGACGGCTTGTTGTGGG	CAACGACGTCTGGAGTTCCA	6.90	6.23
Soltu.DM.02G028380	Ubiquitin-conjugating enzyme E2 I	TCTGCTGCTGCTGGATTTGA	CTTTTTGGCTGCCTGCAACT	-1.73	-1.07
Soltu.DM.03G035910	E3 ubiquitin-protein ligase UBR4	TTGTATGAGGCGACCACCAC	TATCACCACCAGCCTCGAGA	-3.44	-1.38
Soltu.DM.06G020540	E3 ubiquitin-protein ligase UBR4	TTGGCACTGCACTATCCCTG	TGGAAAAAGCCTGGTGGGAA	3.17	2.91
Soltu.DM.03G037450	Ubiquitin-conjugating enzyme E2 I	ATCATCCATGCCAGCTGGAG	TCAAAGCTGAAGCAGAGGCA	2.18	1.50
